# The Claims Data Learning & Enhancing for Algorithm Refinement (CLEAR) Study: Overview of the Study Design and Baseline Profile

**DOI:** 10.2188/jea.JE20250095

**Published:** 2026-06-05

**Authors:** Haruhisa Fukuda, Megumi Maeda, Chieko Ishiguro

**Affiliations:** 1 Department of Health Care Administration and Management, Kyushu University Graduate School of Medical Sciences, Fukuoka, Japan; 2 Laboratory of Clinical Epidemiology, Department of Data Science, Center for Clinical Sciences, Japan Institute for Health Security (JIHS), Tokyo, Japan

**Keywords:** administrative data, diagnostic data, validation studies, disease identification algorithms, Japan

## Abstract

Medical claims data are increasingly used in epidemiological studies but are inadequately validated to ensure their reliability in identifying diseases. We developed the Claims data Learning & Enhancing for Algorithm Refinement (CLEAR) Study as a database platform to enable the systematic, low-cost implementation of validation studies in Japan. The CLEAR Study links routinely generated medical claims data from hospitals with diagnostic data (eg, laboratory data and diagnostic imaging reports) at the patient level. Using diagnostic data as the gold standard for disease identification, researchers can validate and refine their claims-based identification algorithms. Diagnostic data are collected as needed for each validation study, and data are linked using pseudonymized medical record numbers. Personal information is protected through the use of research identification numbers. To demonstrate the platform’s feasibility, we collected data on respiratory syncytial virus (RSV) infections and intussusception cases. Eight hospitals have agreed to participate in the CLEAR Study, and three have completed claims data provision. We obtained data for 5,022 RSV infection cases with 25,920 diagnostic tests, and 1,450 intussusception cases with 561,984 diagnostic tests. We also analyzed the initial diagnostic data for 39,212 RSV infection cases and 439,088 intussusception cases, demonstrating that the database can be used to validate algorithms for these diseases. The CLEAR Study is a newly developed database platform in Japan that facilitates validation studies of claims data for various diseases. By promoting validation studies, this platform will help to improve the reliability of claims-based epidemiological studies and drug risk assessments in Japan.

## PURPOSE

Administrative databases, such as those using insurance claims data, have been developed worldwide, and are increasingly utilized in large-scale epidemiological studies and drug risk assessments.^1^^,^^2^ As these big data repositories involve the routine and automatic collection of patient information generated during health care encounters, they are able to facilitate studies on a wide variety of diseases and treatments without incurring high research costs.^3^^,^^4^ Such databases could be especially useful for research in Japan, as the vast majority of its 120 million population are covered under the universal health insurance system, and all insurers use a standardized claims format. On the other hand, these data are not created for the purpose of research, and it is unclear if they accurately reflect actual clinical conditions. For example, some of the recorded diagnoses in Japanese claims data may not be based on definitive tests but are assigned for insurance billing purposes and to justify investigative procedures. Therefore, validation studies must be conducted to ensure the accuracy of recorded diagnoses in order to minimize measurement bias and increase the research potential of administrative databases.^4^^,^^5^

Many validation studies have been conducted in various countries to examine the accuracy of identification algorithms for specific diseases, such as cardiovascular diseases,^6^ metabolic disorders,^7^^,^^8^ cancers,^9^^,^^10^ and infectious diseases.^11^ These identification algorithms have generally centered around the use of International Classification of Diseases, 10th Revision (ICD-10) codes. However, as healthcare delivery systems and insurance systems vary widely among countries, it is difficult to apply algorithms developed in overseas populations to domestic data.^12^^,^^13^ Despite their necessity, validation studies entail high costs and produce results that can only be applied to epidemiological studies within the country of origin. Accordingly, small research groups may not be able to justify the cost and labor required for such research. While administrative databases are becoming increasingly available and the environment for their application to epidemiological studies continues to improve, there is a burgeoning gap between the use of these databases for research and studies to ensure their validity. Without adequate validation, the results produced by such studies may be inaccurate or skewed. The validation of administrative databases is thought to be especially important in the fields of pharmacoepidemiological studies and post-marketing surveillance.^12^^,^^14^ At present, this lack of validation studies could be considered to be a severe impediment to the advancement of administrative data research in Japan.

In response, we developed the Claims data Learning & Enhancing for Algorithm Refinement (CLEAR) Study as a database platform to enable the systematic and low-cost implementation of validation studies in Japan. The CLEAR Study aims to establish a research environment that facilitates validation studies for a wide range of diseases by linking administrative data with diagnostic data, medical records, and other data types stored by hospitals. Instead of preemptively creating a database with predetermined data elements in anticipation of future research, this platform will collect and provide the data needed by each validation study. In this way, the CLEAR Study will support the development and refinement of disease-specific identification algorithms that are compatible with Japan’s insurance claims system and enable their simultaneous validation at multiple hospitals while minimizing research costs. Herein, we introduce the study design and baseline profile of the CLEAR Study and demonstrate its feasibility by collecting data required to validate the identification algorithms of two etiologically distinct diseases.

## MAIN FEATURES

**Figure 1.**
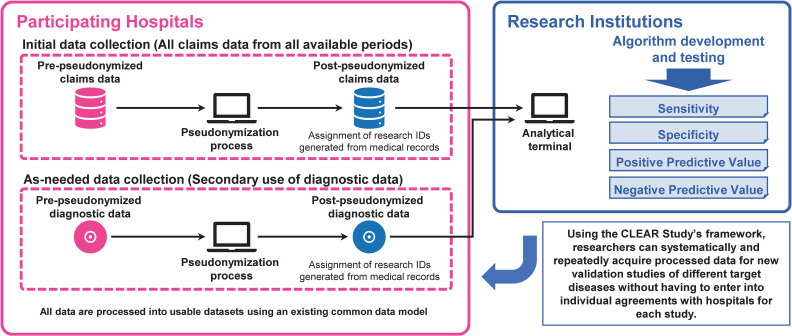
Overview of the CLEAR Study design

The CLEAR Study was designed to collect *all* medical claims data that are generated every month by healthcare facilities for submission to insurance reimbursement agencies. This platform will also collect the relevant diagnostic data (representing the gold standard for indicating the presence of certain target diseases) on an *as-needed basis* for specific diseases in each validation study. To conduct these studies, the claims data are linked with diagnostic data (eg, laboratory data and diagnostic imaging reports) at the patient level (Figure 1). Diagnostic imaging reports refer to those written by radiologists or other physicians. By comparing the recorded diagnoses in the claims data with the diagnostic data, researchers can calculate the sensitivity, specificity, and predictive values of a disease identification algorithm. In addition to claims data and diagnostic data, the CLEAR Study can also acquire other forms of data (eg, medical records and hospital cancer registry data) from participating hospitals when required.

The claims data are obtained from the C-file and D-file of standardized insurance claims that are routinely produced every month by each hospital. These files contain claims for outpatient care, inpatient care, as well as care provided under Japan’s Diagnosis Procedure Combination (DPC) patient case-mix system. In addition, outpatient dispensing data can be collected from the outpatient EF-file of the DPC data. With consent from the participating hospitals, the CLEAR Study has collected medical claims data from as early as April 2008. Claims data from March 2008 and earlier were not collected due to the differences in data items and categories. We obtained claims data on all disease categories and patients during each hospital’s data collection period. While the claims data contain records that diagnostic tests were performed for each patient, they do not include the results of these tests.

In contrast, diagnostic data are not routinely collected from the hospitals. Instead, the necessary data for each validation study’s target disease are requested from participating hospitals on a case-by-case basis. This use of diagnostic data can enable researchers to identify definitive diagnoses for various infectious diseases, such as respiratory syncytial virus (RSV) and coronavirus disease 2019 infections. The target data types of the CLEAR Study can also be expanded to include medical records, adverse event reports, hospital cancer registry data, and Form 1 from DPC data (comprising patients demographic and clinical baseline information) when needed. For validation studies that require information from medical records, the CLEAR Study will extract unstructured text from the hospital-stored medical records containing the relevant clinical information needed for each specific study. The full text of the medical records will not be provided to users.

Data are collected by CLEAR Study staff who directly visit the participating hospitals. All personally identifiable information (eg, names, insurance numbers, and medical record numbers) are converted to hash values in a pseudonymization process. Researchers will determine the participants, outcomes/follow-up and measurements required for each of their studies, and request for these data from the CLEAR Study. The pseudonymized medical claims data and diagnostic data are then released to the researchers to be brought back to their institutions for analysis.

Each patient’s medical claims data are linked with his/her diagnostic data using medical record numbers. Claims data include a registry entry for medical record numbers, which may be encrypted through the use of a secret key. However, applying this key will convert the encrypted values back into the original medical record numbers. Next, participating hospitals are requested to append the medical record numbers to the diagnostic data. At the time of data collection, the CLEAR Study implements its own identification algorithm to the medical record numbers of both the claims data and diagnostic data and sets research identification numbers based on these medical record numbers. These research identification numbers are assigned at the hospital level and are not shared across facilities.

The medical claims data and diagnostic data are consolidated into datasets for study. The claims data are converted into usable datasets based on the Longevity Improvement & Fair Evidence (LIFE) Study’s common data model.^15^ This common data model uses a relational database schema in which each patient’s information (eg, diagnoses, pharmaceuticals, and medical procedures) is separated by tables. Diagnostic data are provided as a single record per test result by the hospitals and can be used in research without the need for standardization. However, the layout of the diagnostic data differs among the hospitals. These test results can be used to guide the development and refinement of claims-based algorithms to approach the gold standard for disease identification.

The CLEAR Study was approved by the Kyushu University Institutional Review Board for Clinical Research (Observational Research: 22305). Information regarding this study is publicly disclosed on the Kyushu University website, and participants are able to opt-out of all CLEAR Study research at any time.

## PARTICIPANTS

The participants of the CLEAR Study will encompass all inpatients and outpatients who have received insurance-covered care at the participating hospitals during the periods for which data are provided (from April 2008). The first milestone of the CLEAR Study is to establish a collaborative system with 10 participating hospitals. At present, eight hospitals have agreed to participate, and three have completed claims data provision. The participating hospitals are distributed across the Kanto, Tokai, Kinki, and Kyushu regions. Researchers who intend to use CLEAR Study data will determine the specific participants (eg, patients within a certain age group) for each individual study.

## OUTCOMES AND FOLLOW-UP

The CLEAR Study’s data platform aims to facilitate validation studies on potentially all diseases that can be accurately identified using each hospital’s diagnostic data. To achieve this aim, the platform is designed to allow researchers to calculate the four main measures of validation studies (sensitivity, specificity, positive predictive value, and negative predictive value) as outcomes. Before using CLEAR Study data, researchers will specify the outcomes (eg, specific diseases) and follow-up period to be used in each individual study. However, the data are hospital-specific, which limits each patient’s follow-up period to the duration in which they receive care at a single hospital.

## MEASUREMENTS

As the CLEAR Study’s data encompasses all claims data, it will enable measurements on various factors, such as patient demographics (age and sex), recorded diagnoses, prescribed medications, medical procedures, surgical procedures, healthcare expenditures, and length of hospital stay. In addition to these claims data, researchers will also acquire corresponding patient-level data that are necessary for the definitive diagnosis of their validation study’s target disease. These diagnostic data include laboratory data, diagnostic imaging reports, medical records, and registry data (eg, cancer registries and Form 1 from DPC data).

## BASELINE CHARACTERISTICS

To demonstrate the feasibility of the CLEAR Study, we collected medical claims data and diagnostic data from three participating hospitals that are needed to conduct validation studies of RSV infections and intussusception. While there were variations in the data collection periods among the hospitals, we obtained claims data from all patients who received care during those periods. Table 1 presents the distribution of patients among the three hospitals. RSV infections were identified using the ICD-10 codes J121, J205, and J210. Intussusception was identified using the ICD-10 code K561.

**Table 1.** Distribution of patients within the claims data of each hospital

		Hospital 1	Hospital 2	Hospital 3
Year	2011	72,079	69,709	—
	2012	75,007	71,477	—
	2013	74,817	73,015	—
	2014	73,529	74,004	—
	2015	73,115	75,966	—
	2016	67,699	76,475	88,914
	2017	69,563	77,247	91,358
	2018	56,263	79,318	86,520
	2019	68,665	80,391	85,035
	2020	56,657	75,773	67,495
	2021	61,270	79,398	72,767
	2022	62,596	81,484	78,445
	2023	39,169	58,406	73,874

Sex	Male	183,077	132,443	123,196
	Female	188,746	157,558	143,015

Age, years	0–19	137,058	50,301	40,800
	20–39	68,695	52,183	52,272
	40–59	60,356	65,604	59,175
	60–79	69,629	91,950	75,552
	≥80	36,085	29,963	38,412

ICD-10 codes	A	80,507	30,649	28,478
	B	34,500	35,222	22,352
	C	24,473	66,662	37,553
	D	51,470	95,186	55,310
	E	115,731	95,028	64,996
	F	30,459	48,567	24,352
	G	54,025	79,413	55,152
	H	53,589	65,603	33,371
	I	68,434	89,621	69,776
	J	158,808	82,762	58,148
	K	129,029	128,769	102,561
	L	63,469	74,239	42,235
	M	84,873	99,587	55,701
	N	57,260	57,288	49,699
	O	10,457	10,336	6,894
	P	7,713	7,246	4,848
	Q	13,303	17,761	5,648
	R	144,905	91,379	107,329
	S	81,605	14,322	38,949
	T	68,265	53,795	32,790
	U	27,447	12,210	31,184
	X	24	7	25
	Z	19,781	26,300	18,293

We acquired the following diagnostic data for RSV infection: simultaneous detection of severe acute respiratory syndrome coronavirus-2 (SARS-CoV-2)/RSV nucleic acid (referral test), simultaneous detection of SARS-CoV-2/RSV nucleic acid (non-referral test), simultaneous detection of SARS-CoV-2/influenza/RSV nucleic acid (referral test), simultaneous detection of SARS-CoV-2/influenza/RSV nucleic acid (non-referral test), RSV antibody titer assay (qualitative, semi-quantitative, and quantitative), RSV antigen assay (qualitative), simultaneous detection of SARS-CoV-2/influenza/RSV antigens (qualitative), simultaneous detection of SARS-CoV-2/RSV antigens (qualitative), simultaneous detection of multiple viral and bacterial nucleic acids (referral test), simultaneous detection of multiple viral and bacterial nucleic acids (non-referral test), and simultaneous detection of multiple viral and bacterial nucleic acids. For intussusception, we acquired data from physicians’ diagnostic imaging reports for ultrasound computer tomography (thoracoabdominal).

From the claims data, we calculated the number of patients with recorded diagnoses of the target diseases as well as the number of patients who had undergone these diagnostic tests. In addition, we also calculated the number of patients according to the results of their initial tests.

### Numbers of patients with the target diagnoses and diagnostic tests

Table 2 shows the numbers of RSV infection patients and intussusception patients from the three hospitals that completed data collection in the CLEAR Study. The table shows the numbers of patients with a recorded diagnosis and who underwent diagnostic tests for each target disease in the claims data. For RSV infection cases, we identified a total of 5,022 patients and 25,920 corresponding diagnostic tests. For intussusception cases, we identified a total of 1,450 patients and 561,984 corresponding diagnostic tests.

**Table 2.** Numbers of patients with the target diagnoses and diagnostic tests

	Data Collection Period	RSV Infection		Intussusception	
	
		Diagnoses^a^	Tests^b^	Diagnoses^c^	Tests^d^
**Hospital 1**	May 2011 to August 2023	4,147	8,975	118	132,854
**Hospital 2**	April 2011 to August 2023	231	1,096	1,063	198,811
**Hospital 3**	April 2016 to February 2024	644	15,849	269	230,319

ICD-10, International Classification of Diseases, 10th revision; RSV, Respiratory syncytial virus; SARS-CoV-2, severe acute respiratory syndrome coronavirus-2.

^a^ICD-10 codes: J121, J205, and J210.

^b^Diagnostic tests: RSV antigen assay (qualitative) (Japanese claims processing code: 160117610), RSV antibody titer assay (160043110), simultaneous detection of multiple viral and bacterial nucleic acids (160216850, 160224150, and 160224050), simultaneous detection of SARS-CoV-2/RSV nucleic acid (160234550 and 160234650), simultaneous detection of SARS-CoV-2/RSV antigens (qualitative) (160234850), simultaneous detection of SARS-CoV-2/influenza/RSV nucleic acid (160235250 and 160235350), and simultaneous detection of SARS-CoV-2/influenza/RSV antigens (qualitative) (160235450).

^c^ICD-10 code: K561.

^d^Diagnostic test: Ultrasound computer tomography (thoracoabdominal) (Japanese claims processing code: 160072210).

### Summary of the initial diagnostic test results

Table 3 summarizes the initial diagnostic test results from the three hospitals that completed data collection. From a total of 39,212 tests performed for RSV infections, 4,473 were positive. From a total of 439,088 patients who underwent thoracoabdominal ultrasound computer tomography, intussusception was detected in 52 cases. These results only demonstrate that the CLEAR Study data can be linked and analyzed, and individual studies are needed to examine the validity of the claims-based algorithms used to identify these diseases.

**Table 3.** Summary of the initial diagnostic test results

	RSV Infection^a^			Intussusception^b^		

	Data Collection Period	Positive	Negative	Data Collection Period	Detected	Not Detected
**Hospital 1**	April 2008 to August 2023	3,649 (4,462)	13,828 (14,433)	April 2008 to October 2023	19	264,933
**Hospital 2**	February 2009 to August 2023	91 (110)	747 (775)	January 2009 to September 2023	1	86,355
**Hospital 3**	April 2016 to March 2024	733 (893)	20,164 (20,273)	April 2010 to February 2024	32	87,748

RSV, Respiratory syncytial virus; SARS-CoV-2, severe acute respiratory syndrome coronavirus-2.

^a^Diagnostic tests: RSV antigen assay (qualitative) (Japanese claims processing code: 160117610), RSV antibody titer assay (160043110), simultaneous detection of multiple viral and bacterial nucleic acids (160216850, 160224150, and 160224050), simultaneous detection of SARS-CoV-2/RSV nucleic acid (160234550 and 160234650), simultaneous detection of SARS-CoV-2/RSV antigens (qualitative) (160234850), simultaneous detection of SARS-CoV-2/influenza/RSV nucleic acid (160235250 and 160235350), and simultaneous detection of SARS-CoV-2/influenza/RSV antigens (qualitative) (160235450).

^b^Diagnostic test: Ultrasound computer tomography (thoracoabdominal) (Japanese claims processing code: 160072210).

Values in parentheses are not limited to the initial test results, but indicate the numbers of patients with positive/negative records.

## STRENGTHS AND LIMITATIONS

In this paper, we introduced the study design and baseline profile of the CLEAR Study, a newly developed database platform designed to facilitate validation studies of claims-based identification algorithms for various diseases in Japan. With cooperation from multiple participating hospitals, this platform could promote validation studies in Japan by linking medical claims data and diagnostic data at the patient level. Furthermore, we demonstrated that the CLEAR Study is able to acquire both data types needed to validate the identification algorithms for RSV infections and intussusception.

Administrative databases, which are generally focused on claims data, have excellent potential to be applied to empirical safety evaluations (eg, post-marketing surveillance of drugs) and drug effectiveness studies in target populations for which randomized controlled trials are not feasible. The United States Food and Drug Administration launched the Mini-Sentinel project in 2009 to monitor medical product safety,^16^ which highlighted the need for validation studies to verify the accuracy of algorithms to identify most outcomes.^17^ As a result, numerous validation studies have been conducted within the Mini-Sentinel project.^2^^,^^18^ Similarly, validation studies have also been conducted using database platforms established by the regulatory authorities of various countries, such as the European Medicines Agency’s Innovative Medicines Initiative PROTECT project,^19^^,^^20^ the European Commission’s EU-ADR project,^21^^,^^22^ the United States Centers for Disease Control and Prevention’s Vaccine Safety Datalink,^23^^,^^24^ Health Canada’s CNODES project,^25^^,^^26^ and the Japanese Pharmaceuticals and Medical Devices Agency’s MIHARI project.^27^

Despite the presence of the MIHARI project in Japan, there is still a lack of validation studies of claims data.^13^ While the number of validation studies conducted domestically has increased in recent years, most are small-scale, single-center analyses.^28^ In 2009, the Japanese government launched the National Database of Health Insurance Claims and Specific Health Checkups (NDB), which collects and stores the claims data and health checkup data of almost all residents throughout the country. Next, a vaccine database containing vaccination records for the entire population is scheduled to be constructed in 2026, and this platform will be designed to integrate with the NDB. However, in contrast to the United States Vaccine Safety Datalink, there are no plans to use the NDB and the national vaccine database for validation studies on vaccine adverse events or vaccine-preventable diseases. Without such studies, there may be a crucial lack of population-based scientific analyses of real-world vaccine effectiveness and safety evaluations within Japan. In order to address this situation, the CLEAR Study was created to provide a database platform that enables validation studies to be conducted at low cost, thereby contributing to the generation of evidence to support the use of administrative data in research. At present, we have completed data collection from three hospitals, including diagnostic data that represent the gold standard for identifying certain diseases. To demonstrate the feasibility of this platform, we collected data on 39,212 RSV infection cases and 439,088 intussusception cases. While the study populations may not meet the criteria for a “perfect” gold standard, the large sample size for each disease is sufficient to obtain a reasonable positive predictive value for validation.^29^

In the context of Japan’s currently available database platforms, the CLEAR Study is characteristic in that it (1) is a multicenter collaborative platform designed with the express purpose of enabling the easy linkage of claims data with diagnostic data at the patient level and (2) provides a research framework in which validation studies for the development of diagnostic algorithms for various diseases can be conducted efficiently and at low cost. At present, there are other databases in Japan that similarly integrate hospital claims data and medical records, such as the Pharmaceuticals and Medical Devices Agency’s MID-NET and the National Hospital Organization’s database.^30^ However, these databases tend to take several months for data extraction, involve high data-usage costs and complex procedures for each dataset, and require on-site processing for individual records. In contrast, the CLEAR Study will provide data for each validation study approximately 1 month after ethics approval is granted. Data will be provided without any usage costs to the researchers.

The CLEAR Study has established a platform that supports the systematic, low-cost implementation of validation studies in Japan. This platform will help to reduce research costs through three mechanisms. First, all claims data from the available periods (ie, the periods for which each hospital agrees to provide claims data) undergo pseudonymization processing during the initial collection. This eliminates the need to repeatedly collect and process additional claims data every time a researcher decides to investigate another disease. In addition, the CLEAR Study uses the LIFE Study’s common data model,^15^ which minimizes data processing costs. Second, all patients are assigned unique research identification numbers generated from their medical record numbers during the pseudonymization process. This allows for the immediate linkage of claims data with the corresponding diagnostic data for each validation study’s target disease. Third, the system involves the secondary use of pre-existing diagnostic data recorded by hospitals, which have agreed to provide such data as needed. Therefore, researchers do not have to conduct new negotiations or enter into new contractual agreements with hospitals whenever they decide to investigate a different disease.

This project has several limitations. First, the CLEAR Study collects medical claims data stored by the participating acute-care hospitals, which do not include claims data from other hospitals or clinics. Therefore, misclassifications may occur when analyzing a patient at a single facility compared to observing him/her across multiple facilities. For example, patients who underwent diagnostic tests at another healthcare facility and whose results were brought to one of the participating hospitals may be missed as false negatives. While insurer-based databases, such as the NDB, enable researchers to determine if diagnostic tests were performed at other healthcare facilities, researchers must exercise caution when considering whether the algorithms developed using the CLEAR Study framework can be applied to other facilities or databases. Second, the gold standard comprises data such as diagnostic data and hospital cancer registry data. While this constitutes the secondary use of pre-existing data produced in the course of clinical care, it is an unavoidable measure to ensure low-cost validation studies. The reliance on such data also means that the CLEAR Study is not suitable for analyzing diseases for which diagnoses cannot be determined based on test results alone. Nevertheless, such studies may be supported through medical record review in the participating hospitals if the additional effort can be invested. Third, the hospitals participating in the CLEAR Study are larger acute-care hospitals (eg, university hospitals) that serve as regional healthcare hubs, and their functions and patient case-mix may not be representative of all healthcare providers in Japan. However, these hospitals are distributed across the Kanto, Tokai, Kinki, and Kyushu regions. As the CLEAR Study supports multicenter validation studies spanning various regions, it could help to increase the generalizability of results by accounting for regional variations. While the CLEAR Study currently has only eight participating hospitals, the vast majority of validation studies for claims data in Japan have been conducted with fewer than five hospitals.^28^ In addition, the use of multiple institutions aligns with the basic principles for validation studies published by the Pharmaceuticals and Medical Devices Agency.^31^

From the perspective of public economics, validation studies can be considered to possess some characteristics of public goods. Publishing the results of validation studies as academic papers enables other researchers to use the validated algorithms with confidence in downstream studies and increases the scope and quality of research using claims data. However, most conventional validation studies involve high costs and labor, which may not justify the production of a single paper. Moreover, many international journals may struggle to find the value in publishing validation studies on Japan’s domestic administrative data, which further disincentivizes such studies. As researchers are free to choose their research topics, these conditions make it difficult for them to invest the time and effort to conduct validation studies. This unfavorable research environment was our main reason for creating the CLEAR Study. With cooperation from several hospitals, the CLEAR Study has already collected and compiled patient claims data into a database. This has allowed us to construct a platform where validation studies can be conducted at will by requesting disease-specific diagnostic data that will serve as the gold standard for testing and refining disease identification algorithms, which greatly reduces the cost and labor required for such studies. We hope that the CLEAR Study contributes to the development of validation studies in Japan by supporting their increased implementation and citation, thereby creating a research environment in which researchers who produce the foundational basis for downstream studies are highly valued. In turn, this could encourage more researchers to conduct claims-based epidemiological studies and drug risk assessments in Japan.
